# Inferior prognosis of gastric involvement in patients with gastrointestinal Burkitt Lymphoma

**DOI:** 10.1002/cam4.2975

**Published:** 2020-03-11

**Authors:** Yi Xie, Mengyu Jia, Jumei Shi, Yi Tao

**Affiliations:** ^1^ Department of Hematology Shanghai Tenth People's Hospital Tongji University School of Medicine Shanghai China

**Keywords:** gastric cancer, lymphoma, prognostic factor, SEER

## Abstract

Due to limited information reported on the clinical characteristics and outcomes of Burkitt lymphoma (BL) patients with gastrointestinal (GI) involvement, here we used the Surveillance, Epidemiology, and End Results (SEER) database to perform our study in a population‐based scale. Extranodal GI involvement was categorized into gastric and intestinal primary sites. A total of 477 BL patients with GI involvement extracted from the SEER database between 2004 and 2015 were included in this study, 112 (23.5%) with the stomach and 365 (76.5%) with the intestine. Our study demonstrated that gastric involvement, older age, male gender, black race, advanced‐stage III/IV, no‐chemotherapy, and earlier years of diagnosis were associated with a significantly worse overall survival (OS) in GI BL patients after adjustment in multivariate analysis, whereas marital status did not significantly influence OS. Notably, BL Patients with gastric involvement had a significantly inferior 5‐year OS in both univariate and multivariate analysis, as compared to those with intestinal involvement (37.8% vs. 70.2%; Univariate: HR = 2.637, *P* < .001; Multivariate: HR = 1.489, *P* = .016). In subgroup analysis, we demonstrated that gastric BL patients had a consistently worse OS than intestinal patients regardless of gender, clinical stage and year of diagnosis. Hopefully, with the advances in modern therapy, improved survival has been found in BL patients with GI involvement as a whole, specifically those with gastric involvement (HR = 0.529, *P* = .011) in recent years of diagnosis. In conclusion, despite the improved survival achieved in recent years, the prognosis of BL patients with gastric involvement is still poor. Novel personalized therapies and better access to intensive care remain to be needed.

## Graphical abstract

Our study included 477 Burkitt Lymphoma (BL) patients with gastrointestinal involvement extracted from the SEER database between 2004 and 2015, 112 (23.5%) with the stomach and 365 (76.5%) with the intestine. We demonstrated that BL patients with gastric involvement had an inferior prognosis compared to those with intestinal involvement (5‐year OS 37.8% vs. 70.2%). In subgroup analysis, gastric BL patients had a consistently worse OS than intestinal patients regardless of gender, clinical stage and year of diagnosis.

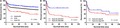

## 1 INTRODUCTION

Burkitt lymphoma (BL) is among the most aggressive B‐cell‐derived non‐Hodgkin lymphoma (NHL) subtypes, which was first described by Dennis Burkitt in the jaw of Ugandan children in 1958. A characteristic event of BL is related to t (8;14) (q24; q32) translocation of c‐Myc and IgH genes, leading to abnormal proliferation of BL cells. Three clinical variants of BL are categorized: the endemic variant, associated with Epstein‐Barr virus (EBV) and occurring primarily in equatorial Africa; the sporadic variant, as the most common variant in the Western world, typically affecting children and young adults worldwide; and immunodeficiency‐associated variant, occurring primarily in human immunodeficiency virus (HIV)‐infected patients.^1^, ^2^, ^3^ These variants are similar in morphology, immunophenotype, and genetic features.

Extranodal involvement is common in BL patients with all three variants. The involvement of the jaws and facial bones is frequently observed in about 50% of patients with endemic BL,^4^ while it rarely occurs in sporadic BL. Sporadic BL accounts for 1%‐2% of adult NHL and 30%‐40% of childhood NHL in Europe and North America.^5^ The most common extranodal sites for sporadic BL are in the abdomen (60%‐80%), especially the ileocecal region. The next most common site is the head and neck, while bone marrow is infiltrated in roughly 20% of patients. The mediastinum, central nervous system, skin, testes, breasts, and thyroid gland are the sites that are rarely involved.^6^ In an Asian study, 72.5% of the BL patients were extranodal at presentation, with the gastrointestinal (GI) tract being the most common site of involvement (27.5%).^7^ BL patients with GI involvement commonly present with abdominal pain, palpable mass, nausea and vomiting, intestinal obstruction, and acute appendicitis.^8^

However, the majority of the available literature describes only single case reports about GI involvement in BL patients. It is unclear the characteristics, prognosis, and risk factors of BL patients with GI involvement in a population‐based scale. In this study, we aimed to provide a better understanding of the characteristics of patients with GI BL, and further compare the outcomes between gastric and intestinal BL in the US, by using the population‐based Surveillance, Epidemiology, and End Results (SEER) database. We also aimed to identify factors that influenced the survival of patients with gastric and intestinal involvement, respectively.

## 2 MATERIALS AND METHODS

### 2.1 Inclusion of patients

Our study was based on data from the US Surveillance, Epidemiology, and End Results (SEER) database. We extracted data of patients with BL diagnosis based on the third edition of the International Classification of Disease for Oncology (ICD‐O‐3) between 2004 and 2015 from the SEER‐18 to conduct this analysis. BL patients with unknown primary sites, clinical stage, or survival time were excluded. The database contained variables indicating age at diagnosis, gender, race, marital status, year of diagnosis, clinical stage, primary anatomical site of involvement, with/without chemotherapy, outcome, survival time, and cause of death. Age was categorized into three groups: 0‐17, 18‐59, and 60 years or older. Race was categorized as white, black, and others. Clinical stage was based on the Ann Arbor staging system. Primary anatomical sites of GI involvement were categorized as stomach and intestine according to the ICD‐O‐3 topography code reported by SEER. Survival time was calculated between the date of diagnosis and the date of death, date last known to be alive, or date of the study cutoff (December 2015), whichever occurs first. Patients were divided into two groups (gastric and intestinal BL) according to the primary anatomical site of BL involvement. Patients with unknown specific gastric or intestinal primary site (n = 12) were not included in further statistical analysis.

### 2.2 Statistical analysis

Statistical analysis was conducted using the Statistical Package for the Social Sciences (SPSS) version 20.0 software (IBM Corporation, Armonk, NY). Proportions of the categorical variables were compared using the chi‐square test. Overall survival (OS) was defined as the period of time from diagnosis to death from any cause. Survival analysis was performed by plotting Kaplan‐Meier survival curves, with hypothesis testing conducted using the log‐rank test. The impact of the categorical variables on the OS was studied by univariate and multivariate analysis using the Cox proportional hazard regression method. A *P*<.05 was considered statistically significant.

## 3 RESULTS

We identified 3487 patients with BL diagnosis recorded in the SEER database between 2004 and 2015. Extranodal involvement (n = 1046) accounted for 30.0% of all cases, while the most common extranodal involvement was gastrointestinal (GI) tract (n = 489, 46.7%). Excluding patients with unknown specific gastric or intestinal involvement (n = 12), this study included a total of 477 patients with GI BL, of which 112 (23.5%) were gastric and 365 (76.5%) were intestinal. The main clinical characteristics in the study cohort are shown in Table 1. The median age at diagnosis of those patients with GI BL was 47 years (range, 2‐94 years) with a male‐to‐female ratio of 4.48:1. Nearly half of them (49.9%) were aged 18‐59. About 81.6% of the included patients were whites, and 40.3% of them were married. The number of patients from 2004 to 2009 was about the same as the number from 2010 to 2015 (n = 241 vs. n = 236, 50.5% vs. 49.5%, respectively). Over half of them were classified as stage I/Ⅱ (59.1%), and the majority of patients (84.5%) received chemotherapy. Further, we compared the characteristics of patients in primary gastric and intestinal sites. There were no statistically significant differences between the two cohorts in terms of gender (*P* = .201), race (*P* = .084), and year of diagnosis (*P* = .576). However, significant differences between the two sites were observed in terms of age categories, marital status, clinical stage, and application of chemotherapy. More patients with gastric BL were elderly (≥60 years) (46.4% vs. 24.7%, *P* < .001), married (44.6% vs. 38.9%, *P* = .004), classified as advanced‐stage III/IV (60.7% vs. 34.8%, *P* < .001), and received no chemotherapy (22.3% vs. 13.4%, *P* = .023), when compared with intestinal BL patients. With a median follow‐up time of 3.7 years, 38.4% of patients were dead. Patients with gastric BL were more likely to die (61.6% vs. 31.2%, *P* < .001), and die of lymphoma (42.9% vs. 20.5%, *P* < .001) than those with intestinal BL.

**Table 1:** Comparison of demographic and clinical characteristics of patients with gastric and intestinal Burkitt Lymphoma

Variable	Total (N = 477)	Primary site		*P* value
		Stomach (n = 112; 23.5%)	Intestine (n = 365; 76.5%)	
Age				
0‐17 y	97 (20.3%)	3 (2.7%)	94 (25.8%)	<.001
18‐59 y	238 (49.9%)	57 (50.9%)	181 (49.6%)	
≥60 y	142 (29.8%)	52 (46.4%)	90 (24.7%)	
Gender				
Female	87 (18.2%)	25 (22.3%)	62 (17.0%)	.201
Male	390 (81.8%)	87 (77.7%)	303 (83.0%)	
Race				
White	389 (81.6%)	86 (76.8%)	303 (83.0%)	.084
Black	45 (9.4%)	10 (8.9%)	35 (9.6%)	
Other	43 (9.0%)	16 (14.3%)	27 (7.4%)	
Marital status				
Married	192 (40.3%)	50 (44.6%)	142 (38.9%)	.004
Single	211 (44.2%)	36 (32.1%)	175 (47.9%)	
Other	74 (15.5%)	26 (23.2%)	48 (13.2%)	
Year of diagnosis				
2004‐2009	241 (50.5%)	54 (48.2%)	187 (51.2%)	.576
2010‐2015	236 (49.5%)	58 (51.8%)	178 (48.8%)	
Ann arbor stage				
Stage I/II	282 (59.1%)	44 (39.3%)	238 (65.2%)	<.001
Stage III/IV	195 (40.9%)	68 (60.7%)	127 (34.8%)	
Chemotherapy				
Yes	403 (84.5%)	87 (77.7%)	316 (86.6%)	.023
No	74 (15.5%)	25 (22.3%)	49 (13.4%)	
Outcome				
Alive	294 (61.6%)	43 (38.4%)	251 (68.8%)	<.001
Dead	183 (38.4%)	69 (61.6%)	114 (31.2%)	
Cause of death				
Lymphoma	123 (25.8%)	48 (42.9%)	75 (20.5%)	<.001
Other causes	354 (74.2%)	64 (57.1%)	290 (79.5%)	

We then evaluated the primary anatomical site, age, gender, race, marital status, year of diagnosis, clinical stage and treatment option in univariate and multivariate models as potential prognostic factors (Table 2). We observed a 5‐year OS of 58.1%, 70.2% and 37.8% for the whole, intestinal and gastric BL diagnosed between 2004 and 2015, respectively. Univariate analysis indicated that patients with gastric BL had a significantly worse OS than those with intestinal BL (HR = 2.637, 95% CI: 1.950‐3.568, *P* < .001). In the multivariate model, primary gastric site (HR = 1.489, 95% CI: 1.078‐2.057, *P* = .016), older age categories (Age 18‐59 y: HR = 8.888, 95% CI: 3.176‐24.870, *P* < .001; Age ≥ 60 y: HR = 17.940, 95% CI: 6.129‐52.514, *P* < .001), advanced‐stage III/IV (HR = 1.826, 95% CI: 1.332‐2.504, *P* < .001), and no‐chemotherapy (HR = 6.138, 95% CI: 4.222‐8.921, *P* < .001) were associated with a significantly worse OS, which was consistent with the conclusion of univariate analysis. However, differing from results of univariate analysis, male gender, black race, and earlier year of diagnosis from 2004 to 2009 were also independently correlated with worse OS after adjustment in the multivariate model, whereas marital status did not.

**Table 2:** Univariate and multivariable prognostic models in patients with gastrointestinal Burkitt Lymphoma

Variable	5‐y OS (%)	Univariate analysis		Multivariate analysis	
		HR (95% CI)	*P* value	HR (95% CI)	*P* value
Primary site					
Intestine	70.2%	1	<.001	1	.016
Stomach	37.8%	2.637 (1.950‐3.568)		1.489 (1.078‐2.057)	
Age					
0‐17 y	95.6%	1	<.001 <.001	1	<.001 <.001
18‐59 y	65.5%	10.111 (3.707‐27.576)		8.888 (3.176‐24.870)	
≥60 y	36.0%	25.834 (9.483‐70.374)		17.940 (6.129‐52.514)	
Gender					
Female	56.1%	1	.079	1	.046
Male	64.2%	0.731 (0.514‐1.037)		1.495 (1.007‐2.222)	
Race					
White	65.4%	1	.161 .020	1	.016 .689
Black	53.1%	1.388 (0.877‐2.197)		1.805 (1.117‐2.917)	
Other	47.7%	1.691 (1.087‐2.630)		1.097 (0.696‐1.729)	
Marital status					
Married	54.0%	1	<.001 .088	1	.554 .754
Single	77.1%	0.434 (0.307‐0.613)		1.124 (0.763‐1.656)	
Other	44.9%	1.375 (0.954‐1.983)		1.066 (0.716‐1.585)	
Year of diagnosis					
2004‐2009	58.6%	1	.156	1	.008
2010‐2015	67.7%	0.805 (0.597‐1.086)		0.659 (0.484‐0.897)	
ann arbor stage					
Stage I/II	71.4%	1	<.001	1	<.001
Stage III/IV	50.2%	2.013 (1.504‐2.694)		1.826 (1.332‐2.504)	
Chemotherapy					
Yes	71.3%	1	<.001	1	<.001
No	14.9%	6.490 (4.709‐8.947)		6.138 (4.222‐8.921)	

Next, we studied the effects of different primary sites on the OS of BL patients in the subgroup analysis (Table 3). We found that patients with gastric BL in the middle age cohort (18‐59 years) had a worse OS than those with intestinal BL (*P* < .001), whereas the other two age cohorts (the 0‐17 and ≥60 years) did not show a similar result. Also, a consistently inferior OS was found in gastric BL patients regardless of gender, clinical stage, and year of diagnosis, with a still worse but seemingly improved trend in recent years 2010‐2015 (*P* = .01) in comparison with the intestinal BL. Conversely, there was no significant difference in OS between the two primary sites in patients of black race (*P* = .104), and in those receiving no chemotherapy (*P* = .173).

**Table 3:** Comparing overall survival of patients with gastric and intestinal Burkitt Lymphoma in different subgroups

Variable	Intestine		Stomach		*P* value
	N	HR (95% CI)	N	HR (95% CI)	
Age					
0‐17 y	94	1	3	0.047 (0.000‐∞)	.806
18‐59 y	181	1	57	2.590 (1.661‐4.038)	<.001
≥60 y	90	1	52	1.327 (0.875‐2.012)	.183
Gender					
Female	62	1	25	2.371 (1.250‐4.495)	.008
Male	303	1	87	2.689 (1.906‐3.793)	<.001
Race					
White	303	1	86	2.492 (1.755‐3.538)	<.001
Black	35	1	10	2.130 (0.855‐5.307)	.104
Other	27	1	16	3.978 (1.628‐9.724)	.002
Marital status					
Married	142	1	50	1.832 (1.185‐2.833)	.006
Single	175	1	36	4.433 (2.498‐7.865)	<.001
Other	48	1	26	1.751 (0.941‐3.258)	.077
Year of diagnosis					
2004‐2009	187	1	54	3.519 (2.376‐5.210)	<.001
2010‐2015	178	1	58	1.876 (1.165‐3.021)	.01
Ann Arbor Stage					
Stage I/II	238	1	44	2.964 (1.839‐4.778)	<.001
Stage III/IV	127	1	68	1.918 (1.287‐2.858)	.001
Chemotherapy					
Yes	316	1	87	2.903 (2.003‐4.209)	<.001
No	49	1	25	1.433 (0.854‐2.404)	.173

**Figure 1:**
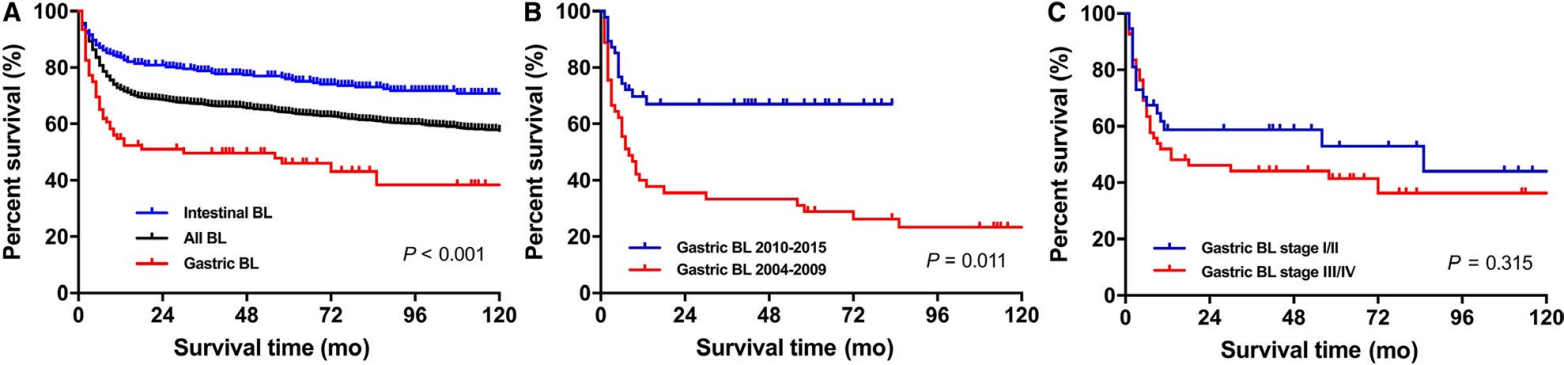
Kaplan‐Meier survival curves of patients with gastric Burkitt Lymphoma (BL)

Survival curves through the Kaplan‐Meier analysis indicated that compared to all BL patients as a whole between 2004 and 2015, patients with gastric BL had a significantly worse OS, while those with intestinal BL had a better OS (Figure 1A, *P* < .001). We further observed the effects of year of diagnosis, clinical stage, and application of chemotherapy on the outcomes of gastric and intestinal BL cohorts, respectively. Interestingly, as shown in Table 4, there was a significant improved survival for patients with gastric BL (HR = 0.529, 95% CI: 0.324‐0.865, *P* = .011; Figure 1B) in the recent era from 2010 to 2015, while little improvement in survival was seen in intestinal patients (HR = 0.961, 95% CI: 0.657‐1.405, *P* = .837). Also, contrary to the intestinal BL, we did not observe changes in the OS between early‐stage I/II and advanced‐stage III/IV subgroup in the gastric BL cohort (HR = 1.290, 95% CI: 0.785‐2.120, *P* = .315; Figure 1C). Expectantly, both gastric and intestinal BL patients benefited from receiving chemotherapy compared to those not receiving (*P* < .001).

**Table 4:** The impact of variables on the overall survival of patients with gastric and intestinal Burkitt Lymphoma

Variable	Stomach		Intestine	
	HR (95% CI)	*P* value	HR (95% CI)	*P* value
Year of diagnosis				
2004‐2009	1	.011	1	.837
2010‐2015	0.529 (0.324‐0.865)		0.961 (0.657‐1.405)	
Ann Arbor Stage				
Stage I/II	1	.315	1	<.001
Stage III/IV	1.290 (0.785‐2.120)		1.941 (1.343‐2.806)	
Chemotherapy				
Yes	1	<.001	1	<.001
No	5.066 (2.970‐8.641)		6.981 (4.653‐10.474)	

## 4 DISCUSSION

Though uncommon, NHL of primary GI involvement accounts for about 30% of extranodal NHL and 4%‐18% of all NHL.^9^, ^10^ However, with a proportion of only 5%, GI involvement of BL variant is a rare presentation among all primary GI‐NHL.^11^ In this study, during the era of 2004‐2015, we identified 489 BL patients with GI involvement from a total of 3487 BL patients, accounting for approximately 47% of extranodal BL and 14% of all BL cases, consistent with GI being the most common extranodal site. Excluding 12 patients with unknown specific primary GI sites, 477 GI BL patients were further categorized into the gastric or intestinal cohort, with a gastric‐to‐intestinal ratio of 1:3.26. In a multivariable analysis of our study, we identified clinical prognostic factors for inferior survival in patients with GI BL, which included gastric involvement, older age, male gender, black race, earlier year of diagnosis from 2004 to 2009, advanced‐stage III/IV, and no‐chemotherapy. GI BL is commonly located in the cecum or small intestine, while primary gastric involvement is extremely rare, representing about 5% of all lymphomas of the stomach and less than 2% of BL,^12^, ^13^ which is consistent with our data of about 3%, with 112 gastric BL out of all 3487 BL patients. Due to its low incidence, in addition to several case reports of gastric BL, there are no large studies published.^11^, ^14^ Gastric BL is mostly diagnosed in middle‐aged persons, but extremely rare in children.^15^ Consistently, our analysis demonstrated only three patients in the 0‐17 age cohort out of 112 gastric BL patients.

In our study, it is essential to emphasize that the gastric involvement was independently associated with inferior survival when compared with the intestinal BL. The 5‐year OS for gastric patients was 37.8%, much inferior to those with intestinal BL 70.2% and the whole BL 58.1%. The significantly lower OS in the gastric could be partially explained by the inclusion of more older patients (46.4% vs. 24.7% of intestinal BL, *P* < .001), more patients with advanced‐stage III/IV (60.7% vs. 34.8% of intestinal BL, *P* < .001), and more patients treated without chemotherapy (22.3% vs. 13.4% of intestinal BL, *P* = .023). However, the negative impact of gastric involvement on OS persists even when adjusted for age, stage, gender, year of diagnosis, and chemotherapy option. In stratified analysis, outcomes in gastric BL were consistently worse regardless of gender, stage, and year of diagnosis, and also in the subgroup of the white race, middle age and patients receiving chemotherapy. This is in contrast to what has been reported in a Korean study demonstrating that gastric BL has superior outcomes to other types of BL.^16^ One possible reason for this disparity is the fact that the Korean study is a small cohort of 21 gastric BL patients, with all of them receiving chemotherapy and approximately half of them (10/21) in early clinical stage I/II. However, despite 71% gastric BL achieving CR in the Korean study, the 2‐year OS was only 55%, which is also inferior to the outcomes of either the intestinal BL (5‐year OS of 70.2%) or the whole BL (5‐year OS of 58.1%) in our study. Interestingly, when focusing on the gastric cohort itself, we found improved survival in the recent era of 2010‐2015 compared to the earlier era of 2004‐2009 (HR = 0.529, 95% CI: 0.324‐0.865, *P* = .011), indicating advances in BL management have translated to outcome improvement in “real world”. However, this improvement was not observed in the intestinal cohort, possibly because of already favorable outcomes obtained for the intestinal BL in the earlier era. In contrast to the intestinal BL, there is no difference in survival between the early and advanced‐stage patients with gastric BL, possibly because the Ann Arbor staging system could not adequately describe the extent of extranodal involvement in the context of gastric patients.

Another concerning finding of this study is the gap between clinical trials and “real‐world” outcomes verified in this study is not the same across age cohorts. Despite its highly aggressive behavior, in the modern era between 2004 and 2015 included in our study when rituximab is widely used, most patients with BL benefited from the use of multiagent intensive chemotherapy combined with rituximab. Long‐term survival has been reported in more than 90% of pediatric patients treated on the Lymphome Malins de *Burkitt* protocols.^17^, ^18^ Consistently, in this population‐based study including 477 patients with GI BL, a seemingly better outcome with the 5‐year OS of 95.6% was observed in patients aged 0‐17 years. However, unfortunately, it has been reported that patients older than 65 years had an inferior prognosis, with a 2‐year OS of 23%.^1^ Similarly, we observed the outcomes for elder patients aged 60 or older were also far inferior, with only 36.0% of patients being alive at 5 years from diagnosis. Despite the inferior prognosis of gastric patients, we have observed no significant difference in survival between the gastric and intestinal involvement in both young and older age cohorts. A contributing factor for the high cure rate in pediatric and adolescent patients may be their preference to participate in therapeutic clinical trials and easy access to better treatments. In contrast, older patients receiving much lower intensity chemotherapy or just palliative care were underrepresented in clinical trials. As for the middle‐aged adults, in a large prospective trial, with the addition of rituximab to intensive chemotherapy, adult patients with BL aged 26‐55 years achieved 5‐year OS of 84%.^19^ Distinctly, our population‐based study showed adult patients with GI BL aged 18‐59 years having a 5‐year OS of 65.5%. The most likely explanation for this distinction is unequal access to rituximab‐based modern immunochemotherapy for patients from clinical trials and “real world”. Notably, our study demonstrated that the distinction was prominent with outcomes in this middle age cohort (18 to 59 years), for gastric patients being much inferior to those with intestinal BL (HR = 2.590, 95% CI: 1.661‐4.038, *P* < .001).

Despite accounting for a small amount of 9.4%, the black race was independently correlated with worse survival in patients with GI BL. The 5‐year OS for black patients was 53.1% compared to 65.4% of white patients. However, there is no significant difference in survival between the gastric and intestinal BL in black patients. Similarly, inferior outcomes for black patients have been reported in other hematological malignancies, such as acute myelogenous leukemia and Hodgkin lymphoma.^20^ This may be partially explained by their inadequate access to modern immunochemotherapy or race susceptibility to treatments,^21^ which could not be confirmed because of unavailable information on specific treatments in the SEER database. As a disease at least in part to be linked to the AIDS epidemic, BL disproportionately affects men. A male‐to‐female ratio of 4.48:1 in GI BL has been reported in our study, higher than the ratio of 2.6:1 in general BL patients,^22^ indicating men with BL are more likely to have extranodal GI involvement.

Of course, there are several limitations inherent in this study. First, SEER do not have access to detailed chemotherapy or immunotherapy in each patient, and these data could have important effects on patient outcomes. Second, information on HIV or EBV status is unavailable, presenting a potential source of bias considering their close association with BL pathogenesis and immune system. In the United States, individuals with HIV are 57 times more likely to develop BL than those without HIV, and approximately 20% of BL patients are living with HIV.^23^ Thus, the specific impacts of HIV infection and the addition of antiretroviral therapy on the outcomes of BL patients with the two different sites were unknown. Additionally, we were also unable to evaluate the effects of laboratory abnormalities or poor performance status on the survival of patients. Studies have been reported that mortality was associated with abnormal lactate dehydrogenase, decreased albumin and poor performance status in children and adult BL patients.^24^, ^25^

Considering the low incidence worldwide, it is meaningful to describe the characteristics and prognostic factors for patients with GI BL in a population‐based scale, especially for extremely rare gastric BL. Our study demonstrated the gastric involvement was an independent inferior predictor for survival in GI BL patients. Hopefully, with the advances in treatment and supportive care, we have observed improved survival in the total of patients with GI BL, specifically those with gastric involvement in the recent year. However, considering the still poor prognosis of patients with gastric involvement, older age and black race, better access to resource‐intense care and tailored immunochemotherapy regimens remain to be urgently needed. Additionally, due to the unavoidable weakness inherent in this study, well‐designed, prospective, and randomized clinical studies should be performed in the future.

## CONFLICT OF INTEREST

All authors disclose no potential conflict of interest.

## AUTHOR CONTRIBUTION

Conceptualization: Yi Tao. Data curation: Yi Tao, Yi Xie and Mengyu Jia. Formal analysis: Yi Tao and Yi Xie. Investigation: All authors. Methodology: All authors. Project administration: Yi Tao. Supervision: Yi Tao and Jumei Shi. Validation: All authors. Writing ‐ original draft: Yi Tao and Yi Xie. Writing ‐ review and editing: All authors.

## Data Availability

The data that support the findings of this study are available from the corresponding author upon reasonable request.
